# Case Report: Mucopolysaccharidosis Type I Treatment With α-L-Iduronidase Replacement Therapy

**DOI:** 10.3389/fped.2022.823044

**Published:** 2022-04-01

**Authors:** Ying Li, Deyun Liu, Yue Yu

**Affiliations:** Department of Pediatrics, The Second Affiliated Hospital of Anhui Medical University, Anhui, China

**Keywords:** mucopolysaccharidosis, mucopolysaccharidosis type I, α-L-iduronidase, laronidase, replacement therapy, case report

## Abstract

Mucopolysaccharidosis is a rare disease and can be divided into seven different subtypes, according to the affected enzyme. Mucopolysaccharidosis type I, the first subtype discovered and reported, mainly affects the *in vivo* storage of degraded sugar. The current treatment methods are symptomatic therapy, enzyme replacement therapy, and allogeneic hematopoietic stem cell transplantation. In China, the enzyme for the treatment of mucopolysaccharidosis type I was approved in June 2020. We report a case of an 18-month-old Chinese boy with mucopolysaccharidosis type I who received enzyme replacement therapy with concentrated laronidase solution. This is the second case of the disease in China, and the first case of a child under 2 years of age. Following the therapy, urine mucopolysaccharide particle levels were significantly lower, and the patient's symptoms improved. The medical records of Chinese patients who have been treated with enzyme replacement therapy for mucopolysaccharidosis type I also showed similar results. This case demonstrated that enzyme replacement therapy is a safe and effective treatment for patients with mucopolysaccharidosis type I.

## Introduction

Mucopolysaccharidosis (MPS) is a group of diseases caused by a genetic mutation, affecting lysosome hydrolase in which mucopolysaccharides cannot be degraded normally; hence, they accumulate in the body, causing a series of symptoms (1, 2). The disease is rare and can be divided into seven subtypes, according to the affected enzyme (3). Mucopolysaccharidosis type I (MPSI) is mainly related to recessive mutations in the *IDUA* gene. The main clinical manifestations include the following: rough facial features, corneal opacity, hepatosplenomegaly, cardiac valve abnormalities, skeletal deformity, lumbar kyphosis or gibbus, and high mucopolysaccharide levels in the urine (4). Enzyme activity analysis is the gold standard for the diagnosis of MPS, and *IDUA* mutation site detection is an important link in MPSI diagnosis (5).

In recent years, international consensus guidelines recommend that patients with MPSI can be classified into three clinical sub-types: Hurler syndrome, Hurler-Scheie syndrome, and Scheie syndrome with the scale of severity being such that Hurler syndrome is the most severe and Scheie syndrome the least severe. The clinical manifestations of each MPS subtype often overlap, making it difficult to distinguish between them during routine examination (6). Data from the international MPSI registry showed that there were a total of 1,007 patients with MPSI as of April 2017 (7). At present, the main treatment methods for MPS are enzyme replacement therapy and allogeneic hematopoietic stem cell transplantation (HSCT) (8). We report the case of the youngest Chinese patient to be diagnosed with MPSI, who was treated with laronidase replacement therapy before HSCT.

## Case Description

The patient was an 18-month-old who was admitted to the hospital due to skin pigmentation with recurrent respiratory tract infection for more than 1 year. The patient is the first child born to healthy, unrelated parents with no family history of MPSI. He was born with grade three amniotic fluid contamination. Three days after birth, he was hospitalized for neonatal hyperbilirubinemia, neonatal pneumonia, and hypoproteinemia. At ~1 month of age, pigment spots appeared on his abdomen, back, buttocks, and lower calves. These spots were pale blue in color; gradually increased in size; and were accompanied by developmental delays, intellectual disability, recurrent respiratory tract infections, and repeated hospital admissions. A cardiac ultrasound diagnosed severe pneumonia and patent ductus arteriosus with pulmonary hypertension, and arterial catheter ligation was performed in another hospital. Genetic examination diagnosed an *IDUA* gene compound heterozygous mutation, and the patient was diagnosed with MPSI.

**Figure 1:**
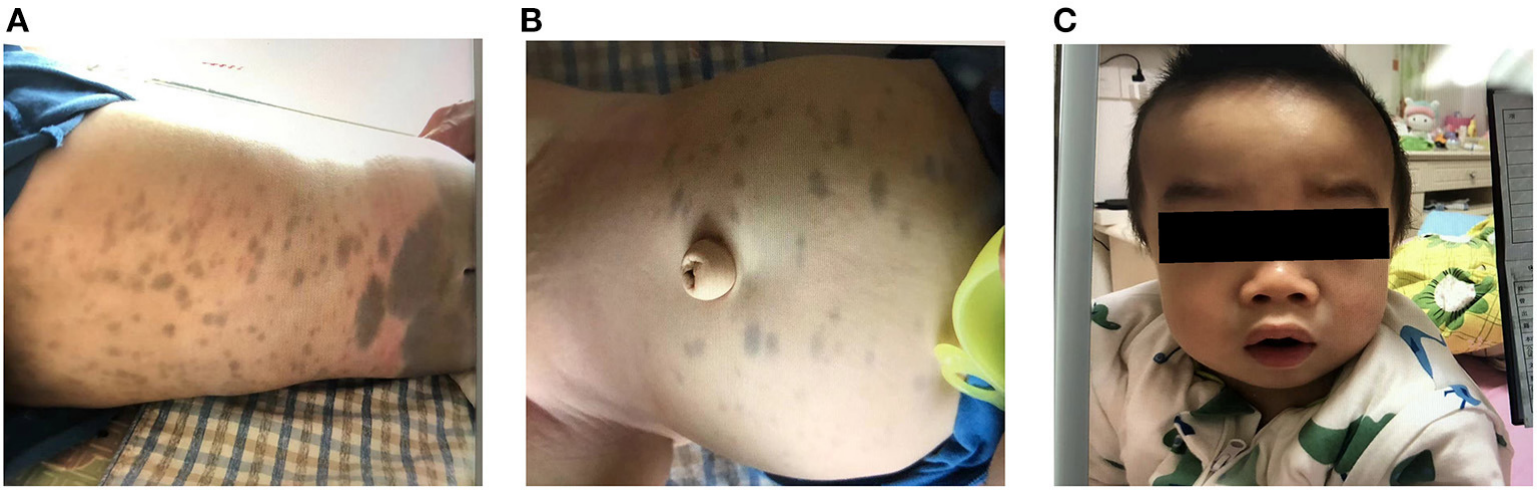
**(A,B)** Large Mongolian spots observed on the abdomen and buttocks of the child. **(C)** The child has thick lips, collapsed nose bridge, nose facing the sky, and enlarged head circumference.

Upon admission, physical examination revealed that the patient could stand and had basic language skills. His abdomen, buttocks, back, and lower calves had visible Mongolian spots. Most of the spots were large and measured ~6 × 6 cm in size, and his head circumference was 51 cm (Figure 1). Assessment of the patient's facial features revealed thick earlobes, collapse of the bridge of the nose, an upturned nose, thick lips, tongue hypertrophy, and a short neck (Figure 1). Respiratory symptoms included mouth and concave breathing with mild costal margin eversion. Abdominal investigations revealed an umbilical hernia. Palpation of the abdomen found a liver span of 4–5 cm from the right costal margin, 2 cm below the xiphoid process, and a 2-cm spleen below the costal margin.

Anteroposterior and lateral radiographs of the whole body's bones and joints showed that all the bones had different degrees of thickening and deformity. A plain chest computed tomography showed that the cervical vertebrae and some thoracic vertebrae were pointy, and the rib bands on both sides had changed, which were consistent with mucopolysaccharide storage disease. Cardiac ultrasound revealed no residual shunt, mild-to-moderate mitral regurgitation, and normal left-sided systolic function of the heart. The distal end of the left main bronchus displayed slight narrowing. Genetic assessments found a heterozygous mutation in the *IDUA* gene complex, c.536C>G, p.Thr179Arg, carried by the patient's mother (possibly pathogenic), and c.1877G>A, p.Trp626Ter, carried by the patient's father (pathogenic), and the patient's α-L-iduronidase level was 0.08 μmol/l (sample type: whole blood; reference interval: 1.91–12.11 μmol/l).

Ibuprofen (5 ml, 20 mg/mL) and levocetirizine (2.5 mg) were administered 1 h before infusion. We prepared for the possibility of allergic reaction or other side effects, which included ensuring that rescue drugs, double venous channels, inhalable oxygen, and suction were available, as well as having equipment for tracheal intubation, tracheotomy and other emergency procedures ready.

After the preparedness, an infusion of 100 ml 0.9% sodium chloride for injection combined with a concentrated solution (1,000 IU) of laronidase (Vetter Pharma-Fertigung GmbH & Co. KG, approval, trade name: Elzan) was administered. According to the drug instruction, the initial rate was 2 ml/h. The infusion rate was doubled every 15 min to a maximum of 32 ml/h. The total infusion time was 3 h and 40 min. During the infusion, the patient's vital signs were stable with normal blood oxygen saturation, blood pressure, temperature, heart rate, and respiration. The patient experienced transient obstructive dyspnea during sleep, although this was relieved with oxygen administration. After the infusion, the vital signs were stable for 24 h, and he was discharged 25 h after the start of the infusion.

**Figure 2:**
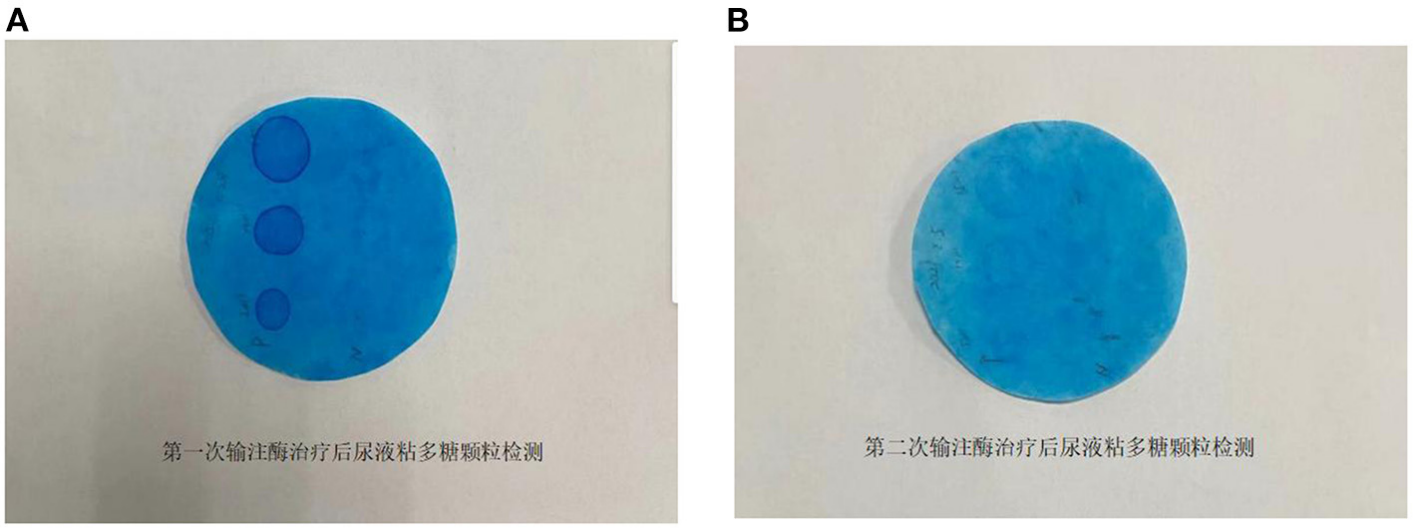
**(A)** Determination of mucopolysaccharide particles in urine after the first enzyme infusion. **(B)** Determination of mucopolysaccharide particles in urine after the second enzyme infusion.

**Figure 3:**
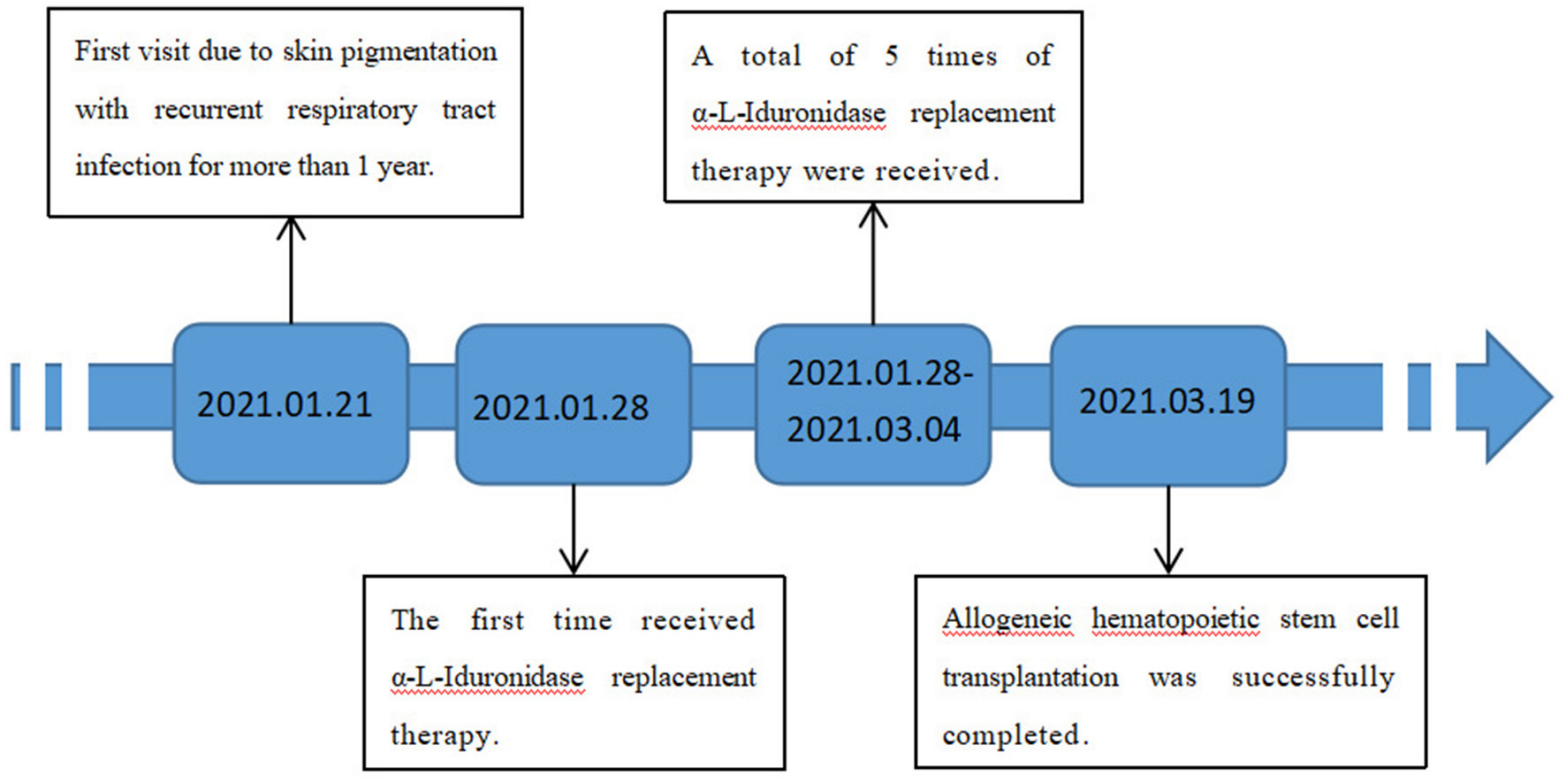
Timeline of the patient's treatment process.

The patient was treated with enzyme replacement therapy weekly until the bone marrow match was achieved (a total of five times), and the allogeneic HSCT was successfully performed on March 19, 2021. After repeating the enzyme replacement therapy, the patient's mucopolysaccharide particle levels in urine were significantly reduced, and the treatment was deemed to have been effective (Figure 2). Therefore, further follow-up visits was not made by the patients. The timeline of the patient's treatment process is shown in Figure 3. Informed consent and full permission for publication of this case report were obtained from the parents.

## Discussion

Mucopolysaccharidosis type I is a defect of lysosomal hydrolases that causes the accumulation of glycosaminoglycan in lysosomes, which leads to facial dysmorphism, bone deformities, hepatosplenomegaly, intellectual disabilities, and multiple organ damage. α-L-Iduronidase replacement therapy is currently the standard treatment for children with MPSI aged older than 18 months. Enzyme replacement therapy using laronidase for MPSI was approved in the United States, Brazil, and China in 2003, 2005, and June 2020, respectively. The first case of laronidase substitution therapy in China was completed in Peking Union Medical College Hospital in 2021, and we report the first case of laronidase substitution therapy in a child under 2 years of age.

Untreated MPSI patients have a lower life expectancy, and before the clinical application of laronidase, the treatment of patients with MPSI was mainly symptomatic therapy and allogeneic HSCT (8). Enzyme replacement therapy is a kind of therapeutic method to achieve a therapeutic effect by exogenous supplementation of enzymes lacking in the body. Studies have shown that HSCT is effective for patients with MPSI, II, IVA, VI, and VII, and the outcome depends on the patient's age; disease severity at the time of surgery; and type of MPS, donor, and treatment regimen (9, 10). Enzyme replacement therapy and HSCT can improve some clinical manifestations of MPS, including range of motion, visual acuity, hearing, cardiopulmonary function, facial characteristic roughness, upper airway obstruction, impaired respiratory function, and hepatosplenomegaly. However, enzyme replacement therapy can improve the physical condition before transplantation and can improve the transplantation success rate. Children with MPSI usually receive enzyme replacement therapy before transplantation to maximize their physical condition pre-transplant, thereby reducing transplant morbidity and risk of death.

Enzyme replacement therapy involves the infusion of deficient enzymes, which circulate only in the blood, have a short half-life, and cannot cross the blood–brain barrier. Enzymes circulating through the blood reduce the accumulation of glycosaminoglycan elsewhere in the body, except in the central nervous system. Hematopoietic stem cell transplantation involves donor stem cells entering the bloodstream, where they can cross the blood–brain barrier and be differentiated in the central nervous system (e.g., into macrophages and microglial cells). Subsequently, the differentiated microglial cells secrete the otherwise deficient enzymes (11, 12). In patients with MPSI, HSCT has been shown to improve central nervous system damage, and the 2009 guidelines for the management and treatment of MPSI listed enzyme replacement therapy combined with HSCT as the gold-standard treatment for children under 2 years of age (5).

The most common adverse reactions associated with α-L-iduronidase replacement therapy are infusion-related reactions (flushing, joint pain, and headache), which were generally mild, although severe allergic reactions were reported in one of 45 patients in a phase III clinical trial (6). Common adverse reactions include rash, nausea, abdominal pain, shortness of breath, bronchospasm, laryngeal edema, respiratory distress, and sleep apnea (commonly observed in MPSI) (13). Therefore, children should be monitored closely for changes in vital signs during α-L-iduronidase infusion. After admission, relevant examinations should be completed, and the patient's condition should be evaluated before enzyme replacement therapy. Multidisciplinary consultation should be conducted with the anesthesiologist, otolaryngologist, and other disciplines, and detailed prevention and emergency plans should be made according to the possible adverse reactions during enzyme replacement therapy and their severity. We believe that the above measures can reduce the incidence of adverse reactions.

In summary, we describe the case of a boy with MPSI from mainland China, who was treated with enzyme replacement therapy, according to the gold-standard modality. The laronidase treatment was a safe and effective treatment before the stem cell transplant. After treatment, the urine mucopolysaccharide particle levels were significantly reduced and the symptoms were relieved. This case was summarized and analyzed to clarify the clinical symptoms and signs of MPSI and to characterize the treatment modality of children with MPSI. Reporting this case adds to the Chinese cases on enzyme replacement therapy treatment in the MPSI medical records and provides a basis for subsequent clinical work and research.

## Patient Perspective

The parents of the children expressed that it was favorable for the children to receive HSCT after treatment, and they were satisfied with the improvement of relevant indicators after treatment.

## Author Contributions

DL and YY were attending physicians of the patient. YL performed the literature review and wrote the first draft of the manuscript. YL and YY assisted in the patient's treatment. DL critically revised the article. All authors contributed to the article and approved the submitted version.

## Conflict of Interest

The authors declare that the research was conducted in the absence of any commercial or financial relationships that could be construed as a potential conflict of interest.

## Publisher's Note

All claims expressed in this article are solely those of the authors and do not necessarily represent those of their affiliated organizations, or those of the publisher, the editors and the reviewers. Any product that may be evaluated in this article, or claim that may be made by its manufacturer, is not guaranteed or endorsed by the publisher.
